# Full time occlusion VS part time occlusion in treatment of monocular amblyopia

**DOI:** 10.12669/pjms.35.6.1287

**Published:** 2019

**Authors:** Mohammad Asim Mehboob, Shoaib Muhammad, Muhammad Asad Farooq

**Affiliations:** 1 Dr. Mohammad Asim Mehboob, FCPS (Ophth), FRCS (Glasg), FICO, MRCSEd (Ophth) Department of Ophthalmology, CMH Gujranwala, Pakistan; 2 Dr. Shoaib Muhammad, FCPS (Ophth) Department of Ophthalmology, CMH Kharian, Pakistan; 3 Dr. Muhammad Asad Farooq, FCPS (Ophth) Department of Ophthalmology, CMH Kohat, Pakistan

**Keywords:** Amblyopia, Full time occlusion, part time occlusion

## Abstract

**Objective:**

To compare improvement in Best Corrected Visual Acuity (BCVA) by Full Time Occlusion (FTO) or Part Time Occlusion (PTO) technique in children with monocular amblyopia.

**Methods:**

This randomized control trial was conducted at Combined Military Hospital, Gujranwala from April 2018 to June 2019. A total of 52 children, diagnosed with non-pathological ametropic amblyopia were randomly divided in two groups. Both underwent cycloplegic refraction and assessment of BCVA. Group A underwent FTO for eight weeks with patch removal only during sleep. Group B underwent PTO for 8 weeks with patching done for six hours a day, out of which 1-2 hours were utilized in near work. Final BCVA was checked at eight weeks, and compared between two groups.

**Results:**

Mean age of study population was 11.06±3.30 years. Mean BCVA before amblyopia treatment was 0.70±0.20 logMAR, and mean BCVA after eight weeks of amblyopia treatment in both groups was 0.29±0.18 logMAR. Difference in BCVA between both groups was statistically significant (p= 0.023). Mean improvement in lines on Snellen’s Visual acuity chart was 1.92±1.35 lines. In our study, 92% of children in FTO group and 66.6% of children in PTO group achieved BCVA of 6/12 or better.

**Conclusions:**

Full time occlusion in children with monocular amblyopia results in greater improvement in BCVA as compared to part time occlusion of six hours per day.

## INTRODUCTION

Ophthalmic diseases in pediatric age group remain under-diagnosed due to a variety of factors including access to health care, availability of reliable medical practitioners, availability of screening facilities and late observation of symptoms and signs by parents. Amblyopia, commonly referred to as “Lazy-Eye” is one such condition, where there is reduced visual acuity in absence of any structural abnormality. The prevalence of amblyopia is approximately 2%, with variation depending on factors like screening facilities, health education and adequate availability of primary healthcare facilities.^1^ Amblyopia is also regarded as the most commonly found cause of visual deterioration in pediatric as well as adults age group.^2^

Amblyopia is due to many reasons, most commonly due to difference in refractive error between two eye (Anisometropic). Other causes of amblyopia are stimulus deprivation due to cataract or any other media opacity, strabismic, in which the decreased vision in lazy eye is due to manifest or latest squint. Other types are ametropic (due to un-corrected refractive error) and meridional (high astigmatism).^3^

Treatment options for amblyopia rely on putting whole visual burden on lazy eye by occlusion or optically negating visual superiority of better eye. This is achieved most commonly by occlusion therapy or pharmacologically achieving cycloplegia of dominant eye.^4^ initially it was believed that amblyopia treatment is effective in young children only, with poor results of occlusion after adulthood. However, now there is evidence advocating need of amblyopia therapy in adults also.^5^

Occlusion therapy still remains the mainstay of treatment for amblyopia. This is done after carefully correcting the refractive error, and then occluding good eye for full time or part time for visual improvement in amblyopic eye. There have been clinical trials comparing Full Time Occlusion (FTO) with Part Time Occlusion (PTO) for management of amblyopia. Some studies indicate the superiority of FTO, while others advocate non-inferiority of PTO.^6^ The exact time and duration for occlusion therapy remains controversial. The objective of this study was to compare improvement in Best Corrected Visual Acuity (BCVA) by FTO or PTO technique in children with monocular amblyopia.

## METHODS

This randomized controlled trial was carried out at Combined Military Hospital Gujranwala from April 2018 to June 2019, after approval from the institutional ethical review committee, and taking written informed consent from parents of patients. A total of 60 children were initially enrolled. Eight patients were lost follow up or didn’t adhere to guidelines strictly, and were therefore excluded from study. A total of 52 children were thus analyzed. Patients from either gender, aged 6-18 years, with normal anterior and posterior segment examination, ametropia confirmed on cycloplegic refraction and BCVA ranging from 6/12 to 6/60 were included in study. Children with history of trauma, congenital glaucoma, congenital cataract, latent or manifest squint, corneal or retinal pathologies, high astigmatism, keratoconus, vernal Keratoconjunctivitis, previous amblyopia treatment and high myopia were excluded. All children underwent detailed ophthalmic assessment, squint assessment with measurement of BCVA. All children underwent fundus examination and cycloplegic refraction after instillation of 1% atropine eye drops. Post mydriatic testing for BCVA was performed two weeks after retinoscopy/fundus examination. After final prescription was given, they were randomly divided in two groups using lottery method. Those in Group A were advised full time patching, with application of tight amblyopia patch on waking up, and removal only before going to bed. Those in Group-B, were advised patching of good eye for six hours a day, out of which, 1-2 hours were mandatory spent on near work. Children were followed up every two weeks for 8 weeks to ascertain the adherence to instructions, measurement of BCVA in good and amblyopic eye, and exclude any other issue. Those failing to comply were excluded from the study. Final BCVA was measured at 8 weeks, and data was entered in the pre devised proforma. Snellen’s visual acuity was converted to logMAR value using online calculators. Confidentiality of the patient’s record was maintained. Statistical Package for Social Sciences (SPSS 20.0) for windows was used for statistical analysis. Descriptive statistics i.e. mean ± standard deviation for quantitative values (age, BCVA) and frequencies along with percentages for qualitative variables (gender) were used to describe the data. We used Shapiro Wilk’s test to check normality of data. Qualitative variables were compared between two groups using Chi Square test and quantitative variables were compared using independent ‘t’ test. A p value of ≤0.05 was considered statistically significant.

## RESULTS

Initially, a total of 60 children were included. Eight children were lost to follow up who were thus excluded. Finally, 52 children were analyzed for this research. Mean age, gender distribution, pre-occlusion BCVA, post-treatment BCVA, and mean improvement in terms of number of lines on Snellen’s visual acuity chart for study population and both groups is given in Table-I. There was no statistically significant difference between two groups in terms of age, gender and pre-occlusion BCVA (p=0.433, 0.726 and 0.945 respectively). Difference in post-treatment mean BCVA between both groups was statistically significant (p=0.023). However, in terms of improvement in number of lines on Snellen’s chart, the difference between both groups was not statistically significant (p=0.158). Frequency of children achieving each BCVA value on Snellen’s chart is shown in Table-II. It is pertinent to mention that in terms of improvement in number of lines read on Snellen’s chart, there was no significant difference between groups. However, logMAR BCVA between both groups showed statistically significant difference.

**Table I:** Clinical Data of Study Population (n=52).

Variable	Total n=52	Group A (FTO Group) n=25	Group B (PTO Group) n=27	p Value (Between groups)
Age (Years) mean ± SD	11.06±3.30	10.68±3.29	11.41± 3.34	0.433*
Gender				
Male	17(65.4%)	9(69.2%)	8(61.5%)	0.560**
Female	9 (34.6%)	4(30.8%)	5(38.5%)	
Pre-treatment BCVA (logMAR) mean ± SD	0.70±0.20	0.70±0.20	0.70±0.21	0.945*
Post-treatment BCVA (logMAR) mean ± SD	0.29±0.18	0.23±0.15	0.35±0.20	0.023*
Improvement in Lines on Snellen’s Chart (no) mean ± SD	1.92±1.35	2.20±1.38	1.67±1.30	0.158*

* Independent ‘t’ Test

** Chi Square test.

**Table II:** Comparison between Two Groups after Treatment.

Variable	Study population n=52	Group A (FTO Group) n=25	Group B (PTO Group) n=27
***Pre-treatment BCVA (Snellen’s value) (%)***			
6/60	5 (9.6)	3 (12)	2 (7.4)
6/48	11 (21.2)	6 (24)	5(18.5)
6/36	9 (17.3)	1 (4)	8(29.6)
6/24	21 (40.4)	13 (52)	8(29.6)
6/12	6 (11.5)	2 (8)	4(14.8)
6/7.5	-	-	-
6/6	-	-	-
***Post-treatment BCVA (Snellen’s value) (%)***			
6/60	-	-	-
6/48	-	-	-
6/36	-	-	-
6/24	11 (21.2)	2 (8)	9(33.3)
6/12	23 (44.2)	12 (48)	11(40.7)
6/7.5	17 (32.7)	10 (40)	7(25.9)
6/6	1 (1.9)	1 (4)	-

## DISCUSSION

Occlusion therapy still remains the gold standard for treatment of amblyopia. We formulated our study based on research and meta-analysis by Yadzani N et al, who recommended that minimum part time occlusion should be at least six hours per day, as patching time less than this does not appear to benefit children will all types of amblyopia.^7^ They also reached conclusion that full time occlusion was comparable to part time occlusion, with no significant difference in final BCVA between both groups. In order to give children in PTO group equal chance for improvement in BCVA based on evidence, the PTO children were patched for 6 hours per day. Also we finalized our study at 8 weeks BCVA results, as it is believed that improvement plateau is reached at 6-8 weeks of patching. In a study conducted by Norris JH and associates, it was recommended that maximum and reliable information about improvement in BCVA is received at six weeks’ time, and patching regimen can be altered or increased depending on that information. They also noted that 14 weeks review does not give more information, as compared to six weeks improvement in BCVA.^8^

We included children with initial BCVA ranging from 6/12 to 6/60, with good Snellen’s chart reading abilities to make research more productive. Younger children, especially pre-verbal have variable response on optotype pictures tests, which would have rendered the study inconclusive. In a study conducted by Arikan G and colleagues evaluating efficacy of occlusion therapy and other factors affecting treatment, it was concluded that initial visual acuity, type of occlusion (FTO/PTO) and initiation age of children undergoing therapy have statistically significant impact on improvement in BCVA, with initial BCVA being the most important risk factor in all kinds of amblyopia.^9^ We did not find any association between initial BCVA and final BCVA, however the results of FTO were superior to PTO, as advocated by Arikan G et al.

In our study, 92% of children in FTO group achieved BCVA of 6/12 or better. However, only 66.6% of children in PTO group achieved BCVA of 6/12 or better. This is in connection with results described by Hug T, who revealed that 67% of children in FTO group, and 46% of children in PTO group achieved BCVA of 20/30 (6/9) or better. They also checked final BCVA after 6 weeks of occlusion therapy. However, they also narrated that in children with initial BCVA of 20/80 or worse, 82% achieved BCVA of 20/40 or better in FTO group and only 40% achieved BCVA of 20/40 or better in PTO group.^10^ These results are in collaboration with results achieved in our study.

Amblyopia has been divided into moderate or severe form, depending on BCVA by recommendations from Pediatric Eye Disease Investigator Group (PEDIG). Moderate amblyopia has BCVA ranging from 20/40 to 20/80, while severe amblyopia ranging from 20/100 to 20/400. In their amblyopia treatment research, it was found that there was improvement of 1.2 lines in children undergoing six hours occlusion, and 0.5 lines in children undergoing two hours occlusion. Improvement of two lines or more was observed in 40% of children undergoing six hours per day occlusion, and 18% of children undergoing 2 hours occlusion.^11^ In another study conducted by Jin YP and associates, children with severe amblyopia underwent 3.9 hours of occlusion, while children with moderate amblyopia underwent 3.2 hours of occlusion. The results on BCVA improvement were comparable between both groups, and thus indicating a shift from FTO to PTO as recommended by PEDIG also.^12^ We feel that improvement in BCVA is dependent on number of hours of occlusion, as stated by PEDIG study, and thus FTO must be superior to PTO, as the improvement of 2.2 lines was seen by us in FTO occlusion group, as compared to 1.67 lines in PTO group.

However, in many studies, PTO has been shown to be effective treatment for moderate as well as severe amblyopia. In study by Irfani I and colleagues, half time occlusion was compared to divided half time occlusion, with results being comparable in both groups.^13^ In another study, the results were similar in FTO and PTO group, but compliance was seen better in children in FTO group. They also recommended FTO in children with PTO failure and poor compliance.^14^ FTO was also considered statistically comparable to PTO in few other studies too.^15^,^16^ Large trials, with good standardization and reliable occlusion compliance may reveal best outcomes for future guidelines.

Other treatment options like atropine penalization and optical penalization have been compared with FTO/PTO for amblyopia. It is believed that atropine and optical penalization, as well as occlusion, either FTO or PTO, have shown equal comparable results and are advocated.^17^,^18^ We recommend that FTO may be initiated as amblyopia therapy in children with severe amblyopia, as it will improve BCVA and also will be compliant for children. While PTO of minimum six hours must be tried in moderate amblyopia, out of which minimum two hours be used for near work.

### Limitations of the study

It includes small sample size, no long term follow up for recurrence of amblyopia and inclusion of children with only anisometropic form of amblyopia. Larger cohorts with inclusion of all types of amblyopia can prove to be more beneficial in terms of advocating a policy guideline for management of amblyopia.

## CONCLUSION

We conclude that full time occlusion is superior to part time occlusion in children with anisometropic amblyopia, with better improvement in best corrected visual acuity, and number of lines improved on Snellen’s visual acuity chart. Also, a greater frequency of patients achieve final visual acuity of 6/12 or better with full time patching, as compared to part time patching. Full time occlusion must be used in children with moderate to severe anisometropic amblyopia for better results and compliance.

## Authors’ Contribution:

**MAM:** Conception, data acquisition takes the responsibility for integrity of research.

**SM:** Did manuscript writing, data analysis.

**MAF:** Did literature review, manuscript editing.
